# Development of Glass Fibers Laminates Toughened with Core–Shell Rubber Particles for Applications in Cold Environments

**DOI:** 10.3390/polym17121641

**Published:** 2025-06-13

**Authors:** Vito Gigante, Bianca Dal Pont, Chiara Montanelli, Laura Aliotta, Andrea Lazzeri

**Affiliations:** Department of Civil and Industrial Engineering, University of Pisa, 56122 Pisa, Italy; vito.gigante@unipi.it (V.G.); bianca.dalpont@phd.unipi.it (B.D.P.);

**Keywords:** toughened laminated composites, vinyl ester urethane resins, core–shell rubber particles

## Abstract

This research focuses on developing high-performance glass fiber laminated composites with improved toughness, particularly for applications in cold environments where traditional composites can suffer from embrittlement and reduced impact resistance. To address this issue, the toughness of Atlac^®^ 580, a bisphenol A-based vinyl ester urethane resin, was enhanced by incorporating core–shell rubber (CSR) particles. Once a mixing procedure to better distribute the CSR particles was identified, the CSR particles were introduced in concentrations ranging from 5 to 15 wt.%. The optimal content for a significant improvement in fracture toughness was identified as 10 wt.%. Finally, three types of glass fiber fabrics with different grammages and weaves were integrated into the optimized resin–CSR system, and their mechanical, morphological, and impact properties were analyzed. The results demonstrated that the toughened resin composite outperformed the reference composite, confirming its enhanced durability and suitability for demanding applications in cold environments.

## 1. Introduction

In highly demanding applications, such as those encountered in the aerospace, automotive, marine, and offshore industries, materials are routinely exposed to harsh chemical and environmental conditions, including low temperatures, humidity, and corrosive agents [1]. In such scenarios, the selection of suitable composite materials becomes critical, not only in terms of mechanical strength and stiffness but also regarding their resistance to crack initiation and propagation [2]. As a result, fracture toughness emerges as a key performance criterion, particularly when structural integrity and long-term durability are paramount [3]. In general, toughness can be described as the ability of a material to absorb energy and deform plastically without fracture, so it requires a combination of both strength and ductility [4].

Traditional thermosetting-based composites, while exhibiting high stiffness and strength, often suffer from intrinsic brittleness, limiting their applicability in critical components under extreme conditions. Addressing this limitation requires developing novel composite systems with enhanced toughness without compromising their mechanical and thermal performance [5].

Analyzing the matrix of this category of composites, thermosets generally have low toughness compared with thermoplastics. This is primarily due to the restricted molecular mobility caused by their cross-linked structure, which results in lower ductility and, consequently, brittle behavior [6]. To overcome this drawback for thermosets, many toughening methods have been studied. In particular, the rubber toughening, and core–shell toughening methods are the most used.

Rubber toughening is a widely adopted strategy to enhance fracture toughness, achieved by incorporating finely dispersed rubber particles, typically in the range of 1–5 µm, into a polymer matrix [7]. These particles are usually formed via reaction-induced phase separation and act as energy absorbers during deformation, thereby delaying or preventing catastrophic failure [8]. The involved toughening mechanisms are diverse and heavily depend on factors such as the chemical structure and composition of the matrix, the morphology of the rubber particles, and the testing conditions [9]: (i) *Microcracking* involves the formation of small, stable cracks near the main crack tip. These microcracks reduce local stress intensity and enhance toughness through a combination of elastic modulus reduction and volumetric expansion [10]. This mechanism has been observed in thermosetting systems such as epoxy resins, where the brittle nature of the cured network makes them susceptible to stress-induced microcrack formation [11]. (ii) *Multiple crazing* prominently occurs in brittle thermosetting polymers, particularly in highly crosslinked epoxies and unsaturated polyester resins. In these systems, rubber particles can induce and stabilize microvoids spanned by fibrils, thereby increasing the material capacity to absorb energy [12]. (iii) *Cavitation* plays a crucial role in enhancing the toughness of thermosetting polymers such as epoxy resins modified with rubber particles [13]. Under high triaxial stress, voids form within the rubber inclusions, relieving hydrostatic stress and facilitating subsequent plastic deformation in the surrounding matrix [14].

Additionally, in rubber-modified epoxy systems, shear bands connecting cavitated rubber particles, called ‘dilatation shear bands’ or ‘croids’, have been observed in the literature and proposed by Lazzeri and Bucknall [15,16,17]. These localized plastic deformation zones arise from void growth and matrix shear yielding around rubber particles, especially under high triaxial stress conditions. Dilatational bands significantly contribute to fracture energy dissipation and have been observed in several thermoset systems, offering an alternative or complementary toughening mechanism to cavitation and multiple crazing.

Rubber toughening, while effective in enhancing fracture toughness, presents several drawbacks. It often leads to reductions in stiffness and tensile strength due to the soft nature of rubber particles. Furthermore, controlling particle size and morphology during phase separation in thermosets can be challenging, and interfacial compatibility issues between the rubber phase and the matrix may further constrain the overall performance. Finally, increased viscosity in the uncured state can hinder processing and fabrication [18].

An advanced strategy to mitigate some of the drawbacks of rubber toughening involves the use of core–shell rubber (CSR) particles. These particles consist of a soft rubbery core (usually butadiene rubber, poly(butyl acrylate), styrene–butadiene rubber, or polysiloxane) encapsulated within a hard, glassy shell (commonly poly(methyl methacrylate), PMMA) [19,20,21]. CSR particles are synthesized through emulsion polymerization, allowing for precise control over particle size and distribution, and they primarily enhance toughness through rubber core cavitation [22]. Importantly, improvements in fracture resistance are directly related to the concentration of CSR particles, with higher concentrations promoting larger plastic deformation zones [23].

Connecting to the aim of this paper, to enhance the toughness of thermoset-based composite materials, this work introduces a novel approach involving a bisphenol-A-based vinyl ester urethane resin matrix modified with core–shell rubber (CSR) particles. To further improve mechanical performance, glass fabrics are employed as reinforcing agents to develop a tougher laminated composite structure.

In the literature, vinyl ester resins with CSR particles used for producing reinforced composites have been investigated [24,25,26]; however, to the best of our knowledge, a vinyl ester urethane system with CSR particles and glass fibers has not yet been explored.

The use of a high-grade bisphenol A-based vinyl ester urethane resin was selected as the matrix for the embedding of the CSR particles because it combines exceptional chemical resistance with a unique balance of thermal stability, high chemical resistance, mechanical strength and hydrolytic stability [27]. In particular, vinyl ester urethane resins are widely employed in marine applications for boat hulls and components due to their balanced performance under harsh conditions, including prolonged exposure to seawater, salinity, and temperature fluctuations [28]. Their molecular structure, derived from epoxy-modified polymers with unsaturated sites, enhances adhesion to reinforcing fibers (e.g., glass and carbon) while resisting osmotic blistering, a critical failure mode in fiberglass composites [29].

It has been studied in the literature that carbon fiber–vinyl ester laminates retain >60% flexural strength after long-term marine exposure, outperforming traditional polyester resins [30]. Moreover, post-curing at 80 °C optimizes crosslinking, achieving glass transition temperatures (Tg) up to 120 °C, which improves dimensional stability under thermal cycling [31].

These resins also offer cost advantages over epoxies while maintaining 80–90% of their mechanical performance, driving adoption in commercial boatbuilding [32]. Ongoing research focuses on optimizing styrene content (30–60 wt.%) to balance viscosity reduction and curing efficiency without compromising environmental resistance [33].

The idea of the work is as follows: Since CSR particles significantly improve the toughness of composite materials by promoting energy dissipation during crack propagation, when incorporated into a vinyl ester urethane matrix, these particles create a three-dimensional microstructure [34] where the particles act as localized zones for deformation, cavitation, and void formation. This mechanism effectively slows crack growth and promotes a transition from brittle to ductile fracture behavior.

CSR particles, when added in concentrations between 1% and 5% by weight to thermoset systems, have been shown to enhance fracture toughness metrics (K_1_c and G_1_c) without compromising the glass transition temperature or mechanical properties [35]. A good and uniform dispersion of CSR particles also facilitates the improved durability and impact resistance of the material [36].

To enhance the toughness of Atlac^®^ 580, a bisphenol A-based vinyl ester urethane thermosetting resin, a customized liquid toughener was developed. This toughener consisted of the same resin mixed with 40 wt.% CSR particles. The study focused on optimizing the mixing process to ensure effective dispersion.

The optimal masterbatch content was determined to be 10 wt.% based on mechanical, rheological, and morphological analyses. To further improve toughness, glass fabrics were used as reinforcement to produce laminated composites.

Three different types of glass fibers were incorporated into the optimized resin–CSR system. The resulting composites were evaluated for mechanical properties, morphological characteristics, and impact resistance, including performance under relevant environmental conditions. These results were compared with those of similar composites made with the same resin and glass fibers but without the addition of CSR particles.

## 2. Materials and Methods

### 2.1. Materials

The materials used as the matrix in this work are listed below:

- Atlac^®^ 580: This is a commercial high-grade bisphenol A vinyl ester urethane resin, provided by AOC resins (Collierville, TN, USA). This resin is used for highly demanding applications since it combines exceptional chemical and heat resistance [density (23 °C): 1.07 g/cm^3^; viscosity: 400–500 mPa∙s].
- Butanox^®^ M-50: This is a medium-reactive methyl ethyl ketone peroxide (MEKP), provided by Nouryon (Amsterdam, The Netherlands), used to cure unsaturated polyester resins [density (20 °C): 1.18 g/cm^3^; viscosity (20 °C): 24 mPa∙s].
- KANE ACE™ MX-010 (K): This is a liquid impact modifier provided by Kaneka (Tokyo, Japan) suitable tailored for improving the toughness of thermoset resins through the dispersion of Kaneka’s novel core–shell rubber (CSR) [viscosity (25 °C): 6000 cPs]. The dispersed core–shell rubber is polybutadiene rubber, and the product contains 40 wt.% of CSR.

The effects of different types of glass fiber fabrics were evaluated to assess the influence of different grammages and weave patterns on the final laminated composite. In particular, the following fabrics were used:

- HEXFORCE^®^: This is a twill glass fiber fabric purchased from Mike Compositi (Milan, Italy). It is used a reinforcement to produce laminated composites. The fabric is woven in twill 2/2 [grammage: 220 g/m^2^; thickness: 1.2 mm].
- Roving 600 111A plain weave glass fiber, produced by Owens Corning (Toledo, OH, USA), is a fabric with a much higher grammage (280 g/m^2^) with respect to HEXFORCE fabric, and the weaving is plain. It is made from Advantex^®^ Glass (Owens Corning, Toledo, OH, USA), a boron-free corrosion resistant E-CR glass fiber.
- M113 chopped strand mat glass fiber produced by Owens Corning (Toledo, OH, USA) is a nonwoven fabric produced using Advantex^®^ glass fibers that combines the mechanical properties of traditional E-glass with the corrosion resistance of E-CR glass. This mat is made with low-size strands of glass roving held together by a styrene-soluble powder binder. The mat grammage is 300 g/m^2^.

**Figure 1:**
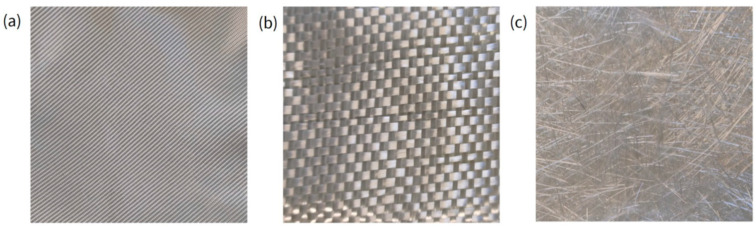
Pictures of (**a**) HEXFORCE^®^, (**b**) Roving 600 111A, and (**c**) M113 chopped strand glass fabrics.

All the glass fabrics were sized with a silane coupling agent. In Figure 1, pictures of the three different typologies of fabric are shown.

### 2.2. Resin Manufacturing and Characterization

The casting method was used to produce a plate of resin from which the samples for mechanical characterizations were produced using the same procedure adopted in [37].

An untoughened resin plate was produced by mixing 98 wt.% of Atlac^®^ 580 and 2 wt.% of Butanox^®^ M-50, as suggested by the supplier, to reduce the gel time. A 24 h cure at 25 °C followed by 3 h of post-cure at 100 °C was performed as reported in the resin technical datasheet. The mixing of the resin with the hardener was carried under vacuum for 2 min using a Polymix mixer (Eurodrop sas, Corsico (MI), Italy).

**Figure 2:**
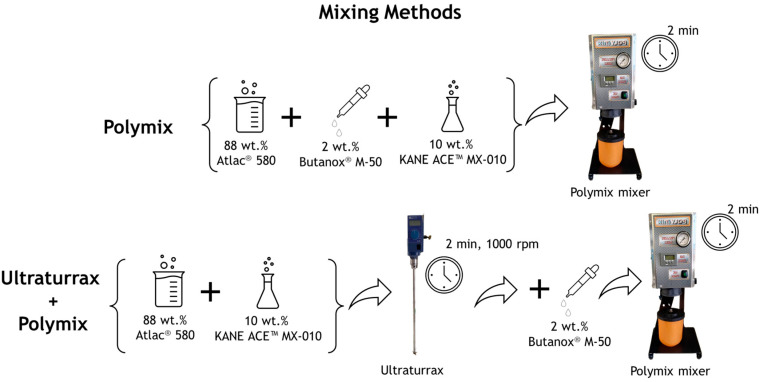
Schematization of the two mixing procedures adopted to disperse the CSR particles.

Two different mixing procedures were investigated to identify the method that achieved the best dispersion of the core–shell rubber particles contained in the KANE ACE™ MX-010 liquid modifier. In both procedures, 10 wt.% of KANE ACE™ MX-010 was combined with the resin and hardener (with the same ratio between the resin and hardener as adopted for the untoughened resin). In the first mixing procedure, an analogue procedure adopted for the untoughened resin was applied using the Polymix mixer and carrying out the mixing under vacuum for 2 min. In the second mixing procedure, the resin and the liquid impact modifier were initially mixed for 2 min in a Ultraturrax agitator (IKA-Werke, Staufen im Breisgau, Germany) at a velocity of 1000 rpm to ensure a higher mixing speed. Subsequently, the hardener was added, and the final mixture was blended under vacuum for 2 min with the Polymix to ensure the removal of any trapped air. The two adopted mixing procedures are schematized in Figure 2.

Once the optimal mixing method had been selected, the effects of the rubber toughener content were investigated. Three compositions were analyzed: 5 wt.%, 10 wt.%, and 15 wt.% of KANE ACE™ MX-010, which corresponded, respectively, to 2, 4, and 6 wt.% of CSR added.

Once the resin plates had been produced according to the procedures mentioned above, the dog bone (ISO 527) [38] and parallelepiped specimen (80 × 10 × 4 mm) needed for the tests were cut from the obtained resin plate using a Charly 4U (CEAST, Turin, Italy) numerical control machine (NCM) interfaced with VisualCut 5.1x22 computer software provided by CEAST (Turin, Italy).

Tensile tests were carried out using an MTS Criterion model 43 universal testing machine (MTS Systems Corporation, Eden Prairie, MN, USA) equipped with an extensometer and a 10 kN load cell. The equipment was interfaced with a computer through MTS Elite Software (version 4.0). Two crosshead speed velocities were settled, the first one at 2 mm/min and the second one at 10 mm/min. The extensometer was removed at a 0.5 mm extension. The grip distance was set equal to 80 mm. Tests were carried out on the resin 48 h after post-curing; during that period, samples were stored in a dry keeper (SANPLATEC Corp., Osaka, Japan) in a controlled atmosphere (room temperature and 50% RH). Five samples were tested for each resin typology.

The flexural properties were evaluated using the above-mentioned MTS apparatus adopting a 3-point bending (3PB) configuration using parallelepiped specimens in the flatwise position. The span distance was 64 mm, and the test was carried out at 2 mm/min.

A minimum of five samples per formulation were tested, and the average flexural modulus and flexural stress were reported. Flexural stress was calculated according to Equation (1):

(1) $$\sigma \;(MPa)=\frac{3PL}{2bd^{2}}$$

where *P* (N) is the maximum load registered before breakage, *L* (mm) is the support span, *b* (mm) is the specimen’s width, and *d* (mm) is the specimen’s thickness. The flexural modulus is calculated according to Equation (2):

(2) $$E\;\left(GPa\right)=\frac{L^{3}m}{4bd^{3}}$$

where *m* (N/mm) corresponds to angular coefficient of the linear elastic part of the load–displacement curve.

Charpy impact tests were performed on a INSTRON CEAST 9050 (Norwood, MA, USA) machine according to ISO 179 [39] on unnotched specimens. At least five samples were used, and the mean Charpy impact strength was evaluated.

To better evaluate the toughening effect, fracture toughness (*K_IC_*) tests were performed on parallelepiped specimens. The aforementioned MTS universal testing (N) machine was used in the 3PB configuration with the specimens in the edgewise position notched with a sharp crack having a length of 5 mm. The crosshead speed was set at 0.5 mm/min, and the span length was set at 40 mm. The fracture toughness was calculated according to Equation (3):

(3) $$K_{IC}\left(MPa\cdot m^{0.5}\right)=\frac{fP}{BW^{1/2}}$$

where *P* (N) is the maximum load registered, *W* (mm) is the specimen’s width, and *B* (mm) is the thickness of the specimen. Parameter f is a geometric factor calculated according to [40].

To select the best mixing method for the dispersion of the liquid impact modifier, a scanning electron microscope (SEM) COMEX EM-30N SEM (Yuseong-gu, Daejeon, Republic of Korea) apparatus was used on the cryofractured surfaces of the specimens. To avoid charge build up, the specimens were first sputtered with gold.

### 2.3. Resin Rheological Evaluation

The viscosity of the toughened and untoughened resins was determined using an RM 100 PLUS viscometer (Champagne-au-Mont-d’Or, France) from Lamy Rheology. A calibration was initially conducted to guarantee the accuracy of the viscosity readings. A MK-SV418 spindle was used. The shear rate, temperature, and duration of the test were set at 20 s^−1^, 25 °C, and 60 s, respectively, for all performed measurements. At least three measurements for each formulation were performed.

### 2.4. Glass Fiber Laminated Composite Manufacturing and Characterization Methods

The number of layers added during the production of the glass fiber laminated composites was determined according to Equation (4) [41]:

(4) $$d=\frac{nA_{w}}{\rho_{f}V_{f}}$$

where *d* (m) is the laminate thickness, *n* the number of fabric plies, *A_w_* (g/m^2^) is the fabric grammage, *ρ_f_* is the fiber density (g/m^3^) and *V_f_* is the fiber volume fraction. Knowing the fiber density (taken from the technical datasheets), maintaining a fixed volume fraction equal to 30% and setting a minimum thickness of 2.5 mm, the number of required plies can be calculated with Equation (4). The number of plies necessary for the different fabrics used is reported in Table 1

To produce the laminated composites, the vacuum bag infusion technique was used. The various glass fiber sheets were then cut into 20 cm wide squares, and their edges were taped off. Subsequently, 22 × 22 cm squares were then cut for both the peel ply and the breather, while the vacuum bag was cut into a 40 × 40 cm square.

Afterwards, the glass layers, the peel ply and the breather ply were stacked and fixed onto a wood plate that was previously thoroughly cleaned and coated with a layer of FR16 release paste wax. Two infusion valves were positioned diagonally. Once the bag was sealed, adhesive tape was placed at the valve points to prevent cuts and breaks, and holes were created using a pointed object. Tubes were inserted for resin infusion and connected to a vacuum pump. The system was checked for vacuum maintenance. Finally, resin was introduced into the inlet tube, and the infusion continued until the fibers were fully saturated. The infusion was conducted at 750 mbar.

Regarding the mechanical characterization of the composites, tensile, impact, and flexural tests were carried out with the same conditions adopted for the resins. For the composite flexural and impact specimens, the same geometry and dimension of the resin specimens were used. For tensile tests, parallelepiped specimens were used instead (110 × 10 × 2.5 mm).

## 3. Results and Discussion

### 3.1. Resin Results Adopting Different Mixing Procedures

**Figure 3:**
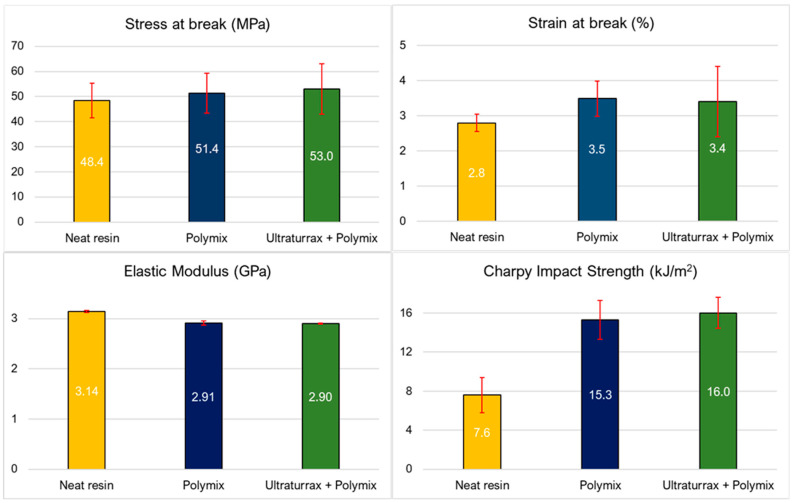
Tensile and impact properties for both rubber toughening mixing methods (the Polymix and the Ultraturrax and Polymix) compared with untoughened resin (neat resin).

Thanks to the mechanical and morphological characterization of the resin toughened with 10 wt.% KANE ACE™ MX-010, it was possible to evaluate which of the two mixing methods—the Polymix mixer alone or a combination of the Ultraturrax agitator and the Polymix—was more effective in dispersing the core–shell rubber (CSR) particles into the resin matrix. In this preliminary screening phase, only tensile and impact tests coupled with SEM analysis were performed to identify the best mixing method. From the mechanical results summarized in Figure 3, it can be observed that both mixing approaches led to a clear improvement in mechanical performance compared with the untoughened (neat) resin. Both stress at break and strain at break noticeably increased. Similarly, the strain at break improved from 2.8% to 3.5% and 3.4%. These enhancements suggest that the addition of CSR particles effectively increased the toughness and slightly improved the ductility of the resin. On the other hand, a slight reduction in the elastic modulus was observed—from 3.14 GPa in the neat resin to approximately 2.90 GPa in both modified systems. This behavior was expected and is consistent with the presence of the softer rubber phase, which reduces overall stiffness but contributes to increased toughness [22].

**Figure 4:**
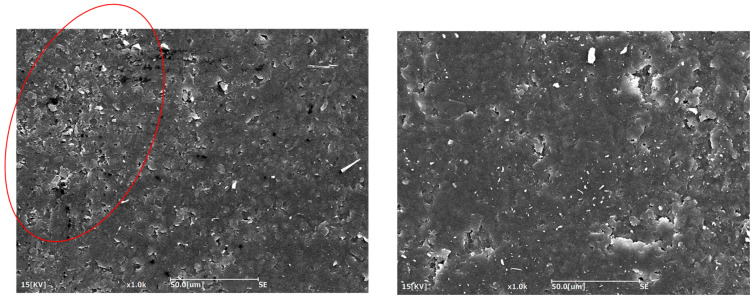
SEM characterization of toughened resin mixed with only the Polymix (**left side**) and with the Ultraturrax and Polymix (**right side**).

The Charpy impact strength also showed a significant increase, more than doubling from 7.6 kJ/m^2^ for the neat resin to 15.3 kJ/m^2^ with the Polymix method and 16.0 kJ/m^2^ with the Ultraturrax and Polymix method. This confirms that both methods were successful in dispersing the rubber phase and enhancing the material’s ability to absorb energy upon impact. However, the slightly higher impact achieved with the Ultraturrax method suggests a more uniform dispersion of CSR particles, likely due to the higher shear forces generated during mixing, which help to break up agglomerates and more evenly distribute the particles throughout the matrix [35]. The better and more uniform CSR dispersion achieved with the combination of Polymix and Ultraturrax was further confirmed by the SEM images reported in Figure 4. The red circle highlights an agglomeration of CSR particles in the mixing procedure in which only the Polymix was used. The agglomeration presence is proof that this was the worst mixing method that, consequently, had a lower toughening effect.

### 3.2. Resin Results with Different Liquid Toughener Amounts

**Figure 5:**
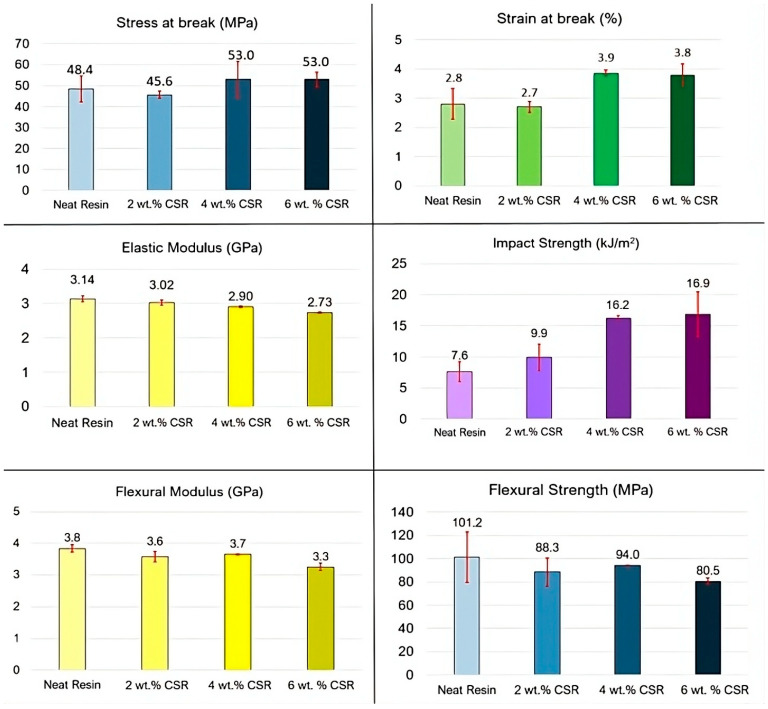
Mechanical properties of untoughened resin (neat) compared with toughened resin with different amounts of CSR particles.

Toughened resins with three different weight percentages of liquid toughener (corresponding to 2, 4 and 6 wt.% of CSR) dispersed with the Ultraturrax and Polymix mixing method were thus investigated. In Figure 5, the results of the mechanical tests are summarized.

The addition of CSR particles increased the strain at break and decreased the elastic modulus of the resin. These results are coherent with the literature [42,43] and are primarily attributed to the lower elastic modulus of the CSR particles compared with the thermoset matrix, which introduces more compliant domains within the material and facilitates energy dissipation during deformation. Regarding the tensile stress at break, no substantial variation was observed with the addition of CSR particles.

Regarding the flexural properties, a decreasing trend could be noticed in both the flexural strength and modulus. The decrement of flexural modulus is coherent with the observed decrement of tensile modulus, and it was related to the presence of the softer CSR phase. On the other hand, the decrement in flexural properties can be ascribed to the fact that the addition of CSR particles introduces compliant regions that absorb energy and increase ductility at the cost of load-bearing capacity under bending stresses [22,44].

Interesting are the results of the Charpy impact strength tests. Increments in the impact properties with CSR content were achieved. The presence of the CSR particles enhanced the impact strength by introducing soft, energy-absorbing domains (that are more prone to cavitation and plastic deformation under load) while also hindering crack growth through pinning and deflection mechanisms. This combination significantly improved the resin’s ability to withstand high speeds impacts [45].

**Figure 6:**
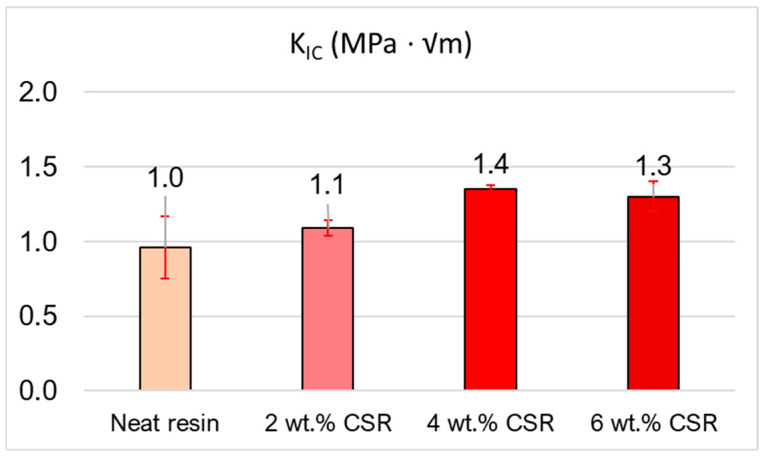
Fracture toughness of neat resin compared with toughened resin with different amounts of CSR particles.

The observed improvement in impact resistance was closely correlated with the registered enhancement in fracture toughness (reported in Figure 6), as both properties stem from the same underlying toughening mechanisms introduced by the CSR. The improvement in the resin fracture toughness enabled the material to better resist crack initiation and propagation under static or dynamic load.

**Figure 7:**
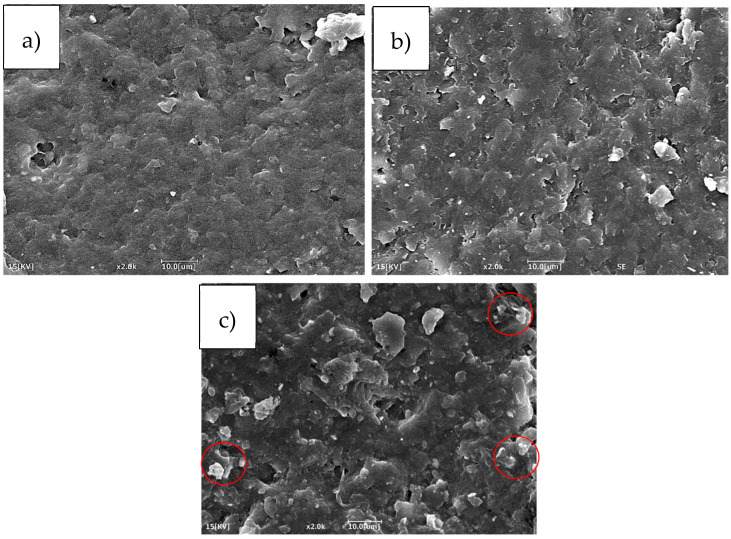
SEM images at a magnification of 2000× of (**a**) 2 wt.% CSR, (**b**) 4 wt.% CSR and (**c**) 6 wt.% CSR.

Nevertheless, it can be observed that no great differences between 4 and 6 wt.% of CSR were present even if the CSR content was increased. This effect was correlated to the fact that increasing the CSR content also increased the probability of creating agglomeration sites. In fact, from the SEM image reported in Figure 7, it can be observed that in the resins with 2 and 4 wt.% of CSR, the overall dispersion of the toughener was quite uniform throughout the samples. On the other hand, increasing the CSR amount up to 6 wt.% resulted in an elevated number of agglomerates, probably due to the higher quantity of present particles (marked with red circles).

**Figure 8:**
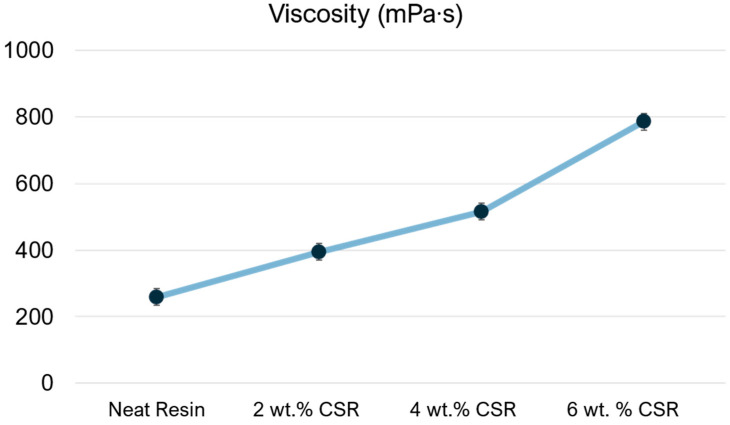
Viscosity values, measured at room temperature, of neat resin compared with toughened resin with different amounts of CSR particles.

Another important aspect to be considered for the selection of the optimal CSR amount is the viscosity change related to its addition. Since the toughened resin will be used to produce glass fiber laminated composites with the vacuum bagging technique, the viscosity must be as low as possible. Infusion-grade resins typically have a viscosity range between 200 and 500 mPa∙s [46]. Generally, CSR addition leads to an increment in viscosity since the presence of these solid particles restricts the flow of resin by creating physical hindrance [47]. In Figure 8, it can be observed that the more particles that were added, the more the resin’s flow was impeded, which led to a marked increment in viscosity.

It was observed that the addition of 6 wt.% CSR resulted in a viscosity increase that could potentially hinder the resin infusion process.

Based on the mechanical and viscosity results, a good balance between mechanical performance and suitable viscosity value was obtained with 4 wt.% of CSR. This amount was used to produce glass fiber laminated composites.

### 3.3. Glass Fiber Laminated Composite Results with Toughened and Untoughened Resin

**Figure 9:**
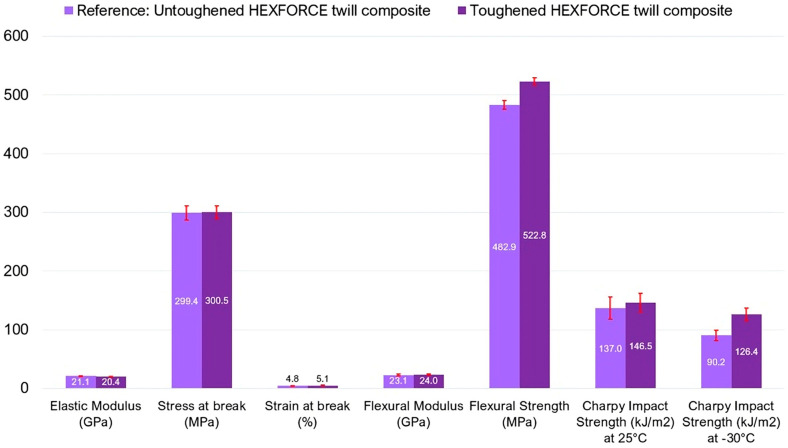
Mechanical behavior of untoughened and toughened composites.

To evaluate the effect of the toughened matrix on the mechanical properties of the composite, a single glass fiber architecture, the twill weave HEXFORCE, was initially selected and used as reinforcement. This setup enabled a direct comparison between the optimized matrix containing 4 wt.% of CSR particles and the unmodified resin without rubber additives. The mechanical characterization of the HEXFORCE^®^ twill glass fiber composites with toughened or untoughened resins as its matrix revealed a slight interplay between the high-performance reinforcement and the rubber-toughened matrix, with implications that varied according to both loading mode and temperature. In Figure 9, it is possible to notice that under uniaxial tension, both the elastic modulus (≈21 GPa) and ultimate strength (≈300 MPa) remained essentially unaffected by the inclusion of 4 wt.% CSR particles, denoting that the stiffness and failure load were overwhelmingly controlled by the glass fibers. Even the slight increase in strain at break (from 4.8% to 5.1%) can be ascribed to localized yielding and cavitation around cavitated rubber cores [48]; however, the rupture process was still dominated by fiber breakage, and the toughening phase contributed little to macroscopic ductility in this regime.

In three-point bending, there were some differences. While the flexural modulus again reflected the fiber-reinforced architecture and remained more or less unchanged (23.1 GPa vs. 24.0 GPa), the flexural strength increases by roughly 8% upon CSR addition. This enhancement indicates that, although bending stiffness is fiber-controlled, crack initiation and propagation in resin-rich interlaminar regions can be significantly hindered by the energy-dissipative mechanisms imparted by CSR particles [49].

The most evident effects of matrix toughening emerged in the impact testing. At 25 °C, the CSR-modified composite absorbed about 7% more Charpy energy than its untoughened counterpart (average values of 146.5 vs. 137.0 kJ/m^2^), indicating a toughening contribution over the glass fiber response. However, when the temperature is lowered to −30 °C, the disparity in performance became glaring: the untoughened composite suffered a 50% reduction in impact energy, reflecting the severe embrittlement of the neat resin, whereas the CSR-toughened system only exhibited a 15% drop. Consequently, the relative impact toughness improvement afforded by the rubber particles swelled to 40%, underscoring the ability of the low-Tg CSR phase to maintain ductile behavior even under subzero conditions.

Taken together, these results highlight a dual-regime behavior: in slow-strain-rate or quasi-static loading (tension and bending), glass fibers dominate and matrix modifications playing a secondary role. Conversely, in high-strain-rate or impact scenarios, particularly at low temperatures, the rubber-toughened matrix has a critical role in energy dissipation and crack arrest. For applications such as offshore or naval structures operating in cold environments where impact resistance and damage tolerance are paramount [50], the incorporation of CSR particles into a glass fiber-reinforced composite offers a clear pathway to improved performance without sacrificing static mechanical properties.

### 3.4. Effect of Architecture, Grammage, Orientation and Type of Glass Fiber Reinforcement

**Figure 10:**
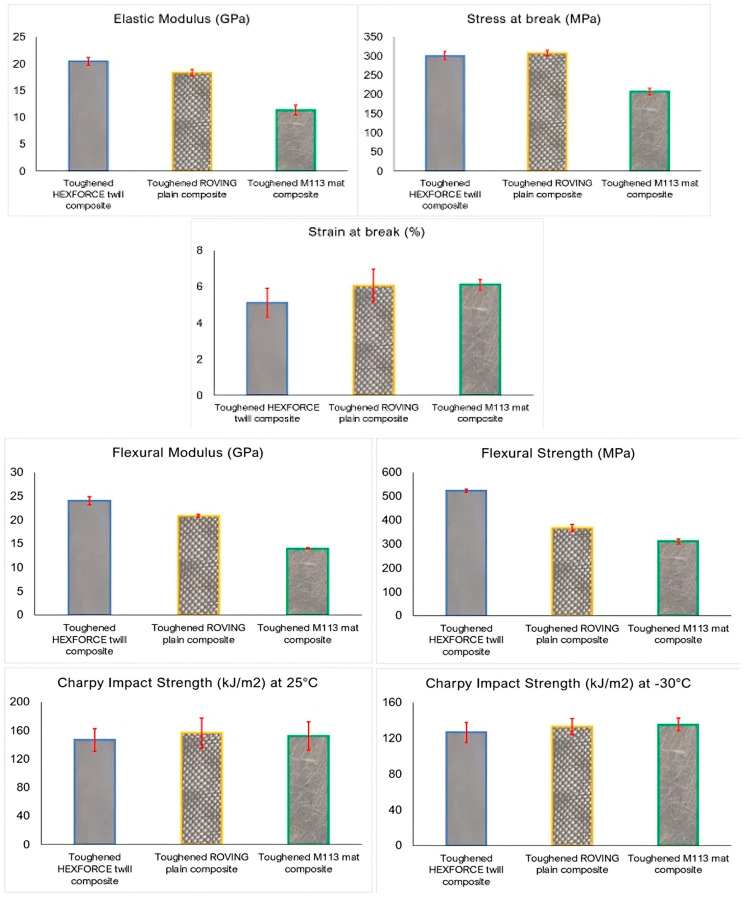
Mechanical properties of composites developed with a toughened resin as the matrix and twill, roving and mat glass fibers as the reinforcement.

To assess how fabric architecture influences the mechanical response of rubber-toughened glass fiber-based composites, three reinforcement types, HEXFORCE^®^ twill, ROVING plain weave, and M113 chopped strand mat, were compared using the optimized toughened resin (4 wt.% CSR) as the matrix. Figure 10 summarizes elastic modulus, tensile strength, strain at break, flexural modulus/strength, and Charpy impact energy at 25 °C and −30 °C.

The chopped strand mat composite exhibited the lowest tensile strength (≈210 MPa) and modulus (≈12 GPa), as the random fiber orientation prevented many filaments from aligning with the load axis, thus reducing load-bearing efficiency [51]. By contrast, both woven architectures (despite different areal weights) yielded comparable stiffness (≈20 GPa) and stress at break (≈300 MPa) values, since most fibers ran parallel to the tensile direction and effectively carry stress [52]. Interestingly, fabric grammage appeared to have negligible impact on the static tensile properties: the M113 mat (highest grammage) still underperformed relative to the lighter woven fabrics.

The flexural stiffness mirrored tensile trends: the HEXFORCE^®^ twill led (≈24 GPa), roving followed (≈21 GPa), and mat trailed (≈14 GPa). The flexural strength, however, was more sensitive to matrix toughening: the tight fiber packing in the twill weave enhanced crack-bridging and limited crack initiation in resin-rich zones, leveraging CSR-induced cavitation and plastic shear to raise the ultimate flexural load [53,54].

Finally, regarding impact properties, at room temperature, the roving plain weave composites produced the highest Charpy impact strength, likely due to its greater areal weight that allowed more fibers to arrest crack growth, while the twill (lowest grammage) absorbed ≈135 kJ/m^2^. The chopped strand mat closely followed the roving weave, reflecting a similar grammage. It can therefore be stated that fabric grammage exerts a dominant influence on the impact resistance of fiber-reinforced composites: they consistently absorb more impact energy than lighter ones. In the literature, even with sandwich structures, panels reinforced with 400 g/m^2^ glass fiber mats have exhibited significantly higher impact strength and energy absorption capacity values than otherwise identical 200 g/m^2^ panels under the same low-velocity impact conditions [55].

Upon cooling to −30 °C, all composites lost toughness, yet their relative ranking persisted. This result is consistent with Putić et al. [56], who showed that fabric areal weight governs impact strength trends even as absolute energies drop at subzero temperatures.

## 4. Conclusions

Impact resistance at low temperatures is a critical performance parameter for glass fiber laminated composites intended for structural applications, especially in environments subject to cold or cryogenic conditions. At reduced temperatures, thermoset polymer matrices typically become more brittle, leading to a higher susceptibility to crack initiation and propagation under impact or sudden loading. This embrittlement significantly compromises the composite’s ability to absorb and dissipate energy, increasing the risk of catastrophic failure.

In this study, the development of toughened laminated composites with enhanced impact properties was performed. The work proceeded through several key steps: First, an optimized mixing procedure was established to incorporate a liquid toughening agent containing core–shell rubber (CSR) particles, ensuring proper dispersion. Then, the effect of varying CSR contents (from 2 to 6 wt.%) was evaluated. It was found that a 4 wt.% CSR amount provided the best compromise between fracture toughness, impact strength and resin viscosity. The latter properties resulted fundamental in the choice of the most suitable CSR content as the toughened resin was compared with an untoughened one for producing glass fiber laminated composites with the vacuum bagging technique.

The addition of the toughened resin on the glass fiber laminates showed a significant improvement in impact properties, as an improvement of 40% of impact strength was registered between the toughened and untoughened composites at −30 °C.

Moreover, in analyzing different glass fabrics, the synergistic effect of fabric grammage, weaving and CSR presence was underlined. The twill fabric demonstrated the best tensile and flexural properties due to the tight pattern in which its fibers are interlaced. On the other hand, the impact properties were found to be dependent on the areal weight of the fabric (grammage); the composite with highest areal weight showed the best impact properties.

Overall, this work offers valuable insights into the combined role of CSR toughening and fabric architecture in overcoming the brittleness of thermoset composites at low temperatures, supporting their potential use in demanding cold-climate structural applications.

## Figures and Tables

**Table 1:** Number of plies for the different glass fabrics calculated according to Equation (3).

Fabric Typology	Number of Plies	Grammage (g/m^2^)	Laminate Thickness (mm)	Glass Fibers Density (g/cm^3^)	Volume Fiber Fraction
HEXFORCE^®^ Twill	9	220	2.5	2.5	0.3
Roving 600 111A Plain Weave	7	280	2.5	2.5	0.3
M113 Chopped Strand Mat	6	300	2.5	2.5	0.3

## Data Availability

The original contributions presented in this study are included in the article. Further inquiries can be directed to the corresponding authors.
